# A Simulation of the Mechanical Testing of the Cell Membrane and Cytoskeleton

**DOI:** 10.3390/mi15040431

**Published:** 2024-03-23

**Authors:** Yue Du, Dai Cheng, Zhanli Yang, Yaowei Liu, Qili Zhao, Mingzhu Sun, Haifeng Li, Xin Zhao

**Affiliations:** 1 The School of Computer and Information Science, Qinghai University of Science and Technology, Xining 810016, China; duyue2007new@163.com; 2 The Department of Computer Technology and Application, Qinghai University, Xining 810016, China; 3 Institute of Robotics and Automatic Information System, The Tianjin Key Laboratory of Intelligent Robotics, Nankai University, Tianjin 300350, China; 2120210429@mail.nankai.edu.cn (D.C.); yzl15649922750@163.com (Z.Y.); liuyaowei@mail.nankai.edu.cn (Y.L.); zhaoqili@nankai.edu.cn (Q.Z.); sunmz@nankai.edu.cn (M.S.); 4 Institute of Intelligence Technology and Robotic Systems, Shenzhen Research Institute of Nankai University, Shenzhen 518083, China

**Keywords:** cell model, DPD, bulk rheology, indentation, cell penetration, cytoskeleton

## Abstract

Cell models play a crucial role in analyzing the mechanical response of cells and quantifying cellular damage incurred during micromanipulation. While traditional models can capture the overall mechanical behavior of cells, they often lack the ability to discern among distinct cellular components. Consequently, by employing dissipative particle dynamics, this study constructed a triangular network-like representation of the cell membrane along with cross-linked cytoskeletal chains. The mechanical properties of both the membrane and cytoskeleton were then analyzed through a series of simulated mechanical tests, validated against real-world experiments. The investigation utilized particle-tracking rheology to monitor changes in the mean square displacements of membrane particles over time, facilitating the analysis of the membrane’s storage and loss moduli. Additionally, the cytoskeletal network’s storage and loss moduli were examined via a double-plate oscillatory shear experiment. The simulation results revealed that both the membrane and cytoskeleton exhibit viscoelastic behavior, as evidenced by the power-law dependency of their storage and loss moduli on frequency. Furthermore, indentation and microinjection simulations were conducted to examine the overall mechanical properties of cells. In the indentation experiments, an increase in the shear modulus of the membrane’s WLCs correlated with a higher Young’s modulus for the entire cell. Regarding the microinjection experiment, augmenting the microinjection speed resulted in reduced deformation of the cell at the point of membrane rupture and a lower percentage of high strain.

## 1. Introduction

Cell micromanipulation involves the precise control of a micropipette’s movement within a microscopic field for manipulating cells or early-stage embryos. This technique encompasses various procedures, including nuclear transfer, microinjection, cytoplasmic extraction, and the introduction of exogenous genes. Emerged in the 1990s, robotic micromanipulation has significantly advanced interdisciplinary research in engineering, life sciences, and biomedical fields [1]. In contrast to manual procedures, robotic micromanipulation plays a pivotal role in standardizing operations, enhancing success rates, and reducing operational costs. Irrespective of manual or robotic micromanipulation methods, cells undergo observable mechanical deformations when subjected to external forces, causing irreversible structural damage to cellular components like the cell membrane and cytoskeleton (e.g., membrane tearing or cytoskeletal fracture) [2,3,4] and significantly impacting cell developmental rates. For instance, despite the advantages of standardized procedures and the high success rates—up to 95%—in robotic nuclear transfer, blastocyst rates remain at around 20%, and the live birth rate is less than 1% [5]. Hence, research in robotic micromanipulation must aim to automate operations while minimizing cell damage.

Studying cell models is crucial to analyzing cellular mechanical responses, quantifying intracellular strain and cell damage, and optimizing operational techniques. Many studies have shown that mechanical strain can affect cell viability and that short-term and high-strain mechanical loading can cause apoptosis. In our prior work, we used internal strain as a criterion for quantifying cell damage in a microinjection experiment [5]. However, we lacked three-dimensional information on cell deformation. The existing methods did not support us in performing the real-time three-dimensional scanning of cells. Although there are methods to calculate cell deformation by injecting fluorescent markers into cells, the latter are damaged in the process. Therefore, developing cell models is an effective tool to analyze cell deformation in three dimensions. Traditional modeling approaches treat cells as continuous entities, employing spring-damping models to simulate cellular responses under varying loading conditions [6,7]. Ref. [6] built an approximate linear model for oocytes utilizing two springs and two dampers; the results, validated against actual experiments, accurately reflected the cell deformation induced by aspiration. However, while suitable for analyzing the microscopic mechanical characteristics of cells, these models cannot differentiate among distinct cell components, each with unique mechanical properties at the microscale. For example, the zona pellucida of oocytes, composed of glycoprotein filaments, is considered to exhibit incompressible elastic properties [8], while the cytoplasm is believed to act as a viscous, non-Newtonian fluid [9]. Therefore, subcellular component modeling serves as a valuable tool for analyzing the mechanical properties of cellular components from an intermediate perspective between the micro- and nanoscales.

Dissipative particle dynamics (DPD) is a mesoscopic simulation method based on statistical mechanics that serves as a bridge between nanoscale and microscale simulations. DPD not only preserves certain atomic details of the system but also overcomes the spatial and temporal limitations of nanoscale simulations. As a result, it provides a valuable theoretical framework for unraveling the mechanical properties of subcellular structures. Prior studies, particularly those involving red blood cells, have extensively focused on modeling the cell membrane. These investigations have primarily considered the phospholipid bilayer structure of the cell membrane while omitting molecular intricacies, yet capturing the microscopic mechanical properties of cells [10,11]. In our preceding work, we characterized the cell membrane as a viscous material and modeled it using a viscoelastic membrane model that considered the viscosity, bending resistance, and incompressibility of the cell. This model involved a triangular mesh, wherein each vertex corresponded to a DPD particle [12].

In contrast, modeling the cytoskeleton poses greater complexity, as it encapsulates the local mechanical characteristics of the cell. The cytoskeleton is commonly perceived as a highly dynamic network of F-actin filaments interconnected by cross-linked proteins. Experimental observations suggest that the behavior of cross-linked F-actin at high oscillation frequencies is primarily governed by thermal fluctuations, leading to the power-law dependency of the storage modulus ($G^{\prime }$) and loss modulus ($G^{\prime \prime }$) on the frequency. Furthermore, the network displays an elastic response at low frequencies. The authors in [13] analyzed the dynamics of transient cross-linking networks, proposing a semi-phenomenological approach to elucidating network behavior at low to medium frequencies. Another study [14] simulated a network comprising individual actin monomers and actin cross-linking proteins (ACPs) using Brownian dynamics. This analysis focused on unraveling the unfolding patterns of cross-linking proteins and assessing the impact of diverse constraints on network responses. However, existing simulation studies still grapple with achieving a dynamic cytoskeletal network that accurately mirrors the intricate rheological responses observed in experiments and replicates the viscoelastic properties evident in real-world scenarios.

Parameter determination and model validation play pivotal roles in cell modeling, often necessitating cell mechanics measurement experiments. These experiments frequently encompass techniques such as Atomic Force Microscopy (AFM)-based nanoindentation, micropipette aspiration, microrheology, microfluidics, magnetic tweezers, or optical tweezers. For instance, in nanoindentation experiments, force–indentation curves are obtained and fitted to determine the Young’s modulus of the simulated cell. Another critical validation experiment is microinjection. Constructing the simulation environment for microinjection allows for the observation of cell deformation at varying penetration speeds, with intracellular strain being commonly calculated as a cell damage indicator.

In this study, we developed a cell model using DPD which comprises a triangular network-like representation of the cell membrane and cross-linked cytoskeletal chains. We scrutinized the mechanical properties of both the membrane and the cytoskeleton through a series of simulated experiments, which were subsequently validated against physical experiments. Initially, to analyze the membrane’s storage and loss moduli, a particle-tracking rheology experiment was conducted by monitoring the mean square displacements of membrane particles over time. Subsequently, a double-plate oscillatory shear experiment was employed to assess the storage and loss moduli of the cytoskeletal network. The simulation results indicate that both the membrane and the cytoskeleton exhibit viscoelastic behavior, as demonstrated by the power-law dependency of their storage and loss moduli on frequency. Furthermore, we conducted indentation and microinjection simulations mimicking real experiments to analyze the mechanical properties of whole cells, comprising the simulated cell membrane and cytoskeleton. In the indentation experiment, forces were recorded as the probe approached the cell, facilitating the derivation of force–indentation curves for the determination of Young’s modulus. Additionally, we investigated the impacts of varying membrane parameters, including the shear modulus ($\mu_{0}$), the bending stiffness ($k_{bending}$), and the maximum length ($l_{max}$), on the force–indentation curves. For the microinjection experiment, we constructed a DPD environment mirroring the actual microinjection platform, featuring a holding micropipette, a cell, and a microinjection micropipette. The analysis centered on observing cell deformation at different microinjection speeds. The intracellular strain distribution within the simulated cell was plotted and compared with visually observed results from the actual experiment, utilizing metrics like Jensen–Shannon (JS) divergence, the Jaccard similarity coefficient, and Bhattacharyya distance.

The remainder of this paper is structured as follows: Section 2 describes the DPD method and the cell membrane and cytoskeleton models. This section elucidates the simulated mechanical testing conducted on these two components, including the theoretical framework behind particle-tracking rheology for the membrane and the bulk rheology approach for the cytoskeleton. Additionally, it outlines the setup procedures for two distinct cell mechanics models—the indentation model and the cell microinjection model—alongside the setup protocols for the actual experiments associated with these models. Section 3 details the outcomes derived from the particle-tracking rheology of the membrane, focusing on the mean square displacements (MSDs) of membrane particles over time, along with the determined storage and loss moduli of the membrane. It also presents the analytical findings related to the bulk rheology of the cytoskeletal network, which were derived from the stress curves obtained at varying shear strain frequencies. Moreover, this section analyzes the stress amplitude and phase difference concerning the applied shear strain. Finally, it includes discussions on the results stemming from the indentation experiment, delineating the force–indentation curves associated with different critical membrane parameters, and the outcomes of the microinjection experiment, focusing on the cell deformation observed at different microinjection speeds. Section 4 discusses the findings of the study, while Section 5 serves as the conclusion of this paper.

## 2. Materials and Methods

### 2.1. Dissipative Particle Dynamics and Cell Model

#### 2.1.1. Dissipative Particle Dynamics

The DPD method, a mesoscopic statistical mechanics approach, finds widespread use in simulating intricate fluids and biological tissues. This system ensures momentum conservation and adheres to Newton’s equation of motion. Within the DPD framework, individual particles represent multiple molecules, and interactions among non-bonded particles are categorized into three types: conservative force ($F_{ij}^{C})$, dissipative force ($F_{ij}^{D}$), and random force ($F_{ij}^{R}$). These interactions operate solely within the defined cutoff radius ($r_{c}$). The conservative force embodies a soft repulsion and is mathematically expressed as

(1) $$F_{ij}^{C}=\left\{\begin{array}{cc} a_{ij}(1-\frac{r_{ij}}{r_{c}})\hat{r_{ij}} & r_{ij}<r_{c}\\ 0 & r_{ij}\geq r_{c} \end{array}\right.$$

where ${\hat{r}}_{ij}=r_{ij}/\left|r_{ij}\right|$ represents the directional vector between both particles and $a_{ij}$ is the repulsion strength between particles *i* and *j*. The dissipative and random forces, which are interlinked via the fluctuation–dissipation theorem, are defined as

(2) $$F_{ij}^{D}=-\gamma \omega^{D}(r_{ij})<v_{ij},{\hat{r}}_{ij}>{\hat{r}}_{ij}$$

where $\omega^{D}$ is a weight function that describes the decreasing dissipative force as the distance between the particles increases, $v_{ij}=v_{i}-v_{j}$ describes the relative speed between the two particles, and γ is the friction coefficient.

(3) $$F_{ij}^{R}=\sigma \omega^{R}\left(r_{ij}\right)\frac{\epsilon_{ij}}{\sqrt{dt}}\hat{r_{ij}}$$

where σ is the random force coefficient, $\omega^{R}$ and $\omega^{D}$ are weight functions related to distance $r_{ij}$, $\epsilon_{ij}$ is a random variable following a normal distribution with a zero mean and unit variance $\epsilon_{ij}=\epsilon_{ji}$, and the directions of dissipative force $F_{ij}^{D}$ and random force $F_{ij}^{R}$ are also along the directional vector between particles *i* and *j*. These weight functions must satisfy the condition

(4) $$\begin{array}{cc} \omega^{D}\left(r_{ij}\right)={\left[\omega^{R}\left(r_{ij}\right)\right]}^{2} & =\left\{\begin{array}{cc} {\left(1-r_{ij}\right)}^{2}, & r_{ij}<r_{c}\\ \;0, & r_{ij}\geq r_{c} \end{array}\right.\\ \sigma^{2} & =2\gamma k_{B}T \end{array}$$

#### 2.1.2. Cell Membrane

The cell membrane primarily consists of a phospholipid bilayer, housing various types of membrane proteins integrated onto its surface. In this model, the cell membrane is depicted as a triangular network [15], and the vertices are interconnected through worm chains (WLCs).

This model considers several factors: the elastic energy ($U_{elastic}$), the bending resistance ($U_{bending}$), and the volume ($U_{volume}$) and surface area ($U_{area}$) constraints. Additionally, it incorporates a dissipative force and a random force applied to the springs connecting two particles, aiming to describe the viscoelastic nature of the membrane [12,16].

(5) $$U=U_{elastic}+U_{\mathrm{bending}\;}+U_{\mathrm{area}\;}+U_{volume}$$

The shear modulus ($\mu_{0}$) of each WLC is expressed as

(6) $$\mu_{0}=\frac{\sqrt{3}k_{B}T}{4pl_{\max}x_{0}}\left(\frac{x_{0}}{2{\left(1-x_{0}\right)}^{3}}-\frac{1}{4{\left(1-x_{0}\right)}^{2}}+\frac{1}{4}\right)+\frac{\sqrt{3}k_{p}\left(m+1\right)}{4l_{0}^{m+1}}$$

where $x_{0}$ is the ratio of the spring’s equilibrium length to the maximum length ($\frac{l_{0}}{l_{\max}}$), $k_{p}$ is the spring constant, *m* is a user-specified exponent, *p* is the persistence length, and $k_{B}T$ is the energy unit. *p* and $k_{p}$ control the overall stiffness of the cell membrane and are related to $\mu_{0}$ and $l_{max}$.

#### 2.1.3. Cytoskeleton

The cell cytoskeleton comprises three primary classes of elements—microtubules, actin filaments, and intermediate filaments—differing in size and protein composition. With the involvement of various auxiliary proteins, these filaments interconnect to create a complex three-dimensional cytoskeletal network, significantly influencing both the mechanical properties and the biological functions of a cell. In our approach, we aimed to replicate its mechanical phenomena rather than delving into specific biomechanical reactions or interpreting cellular characteristics at a molecular level. Within the model, each filament represents real filament bundles, without differentiation among actin filaments, microtubules, and intermediate filaments. Each filament is composed of particles connected by harmonic bonds. These filaments are cross-linked via actin cross-linking proteins (ACPs). Each particle on the filaments can bind with an ACP, while an ACP can link up to two filament particles. Adjacent particles on the filaments are restricted by two harmonic potentials:

(7) $$\begin{array}{cc} U_{bond} & =k_{bond}{\left(r-r_{0}\right)}^{2}\\ U_{angle} & =k_{angle}{\left(\theta -\theta_{0}\right)}^{2} \end{array}$$

where $k_{bond}$ is the spring constant; $k_{angle}$ is the bending stiffness; and *r* and θ are the length of a spring and the angle between two springs, respectively. In addition to the two-body potential and three-body potential regulated by the harmonic law, the interaction between the filament particles and ACP particles involves a four-body potential, utilized to portray the twist between two interconnected filaments mediated by an ACP.

(8) $$\begin{array}{c} U_{bending}=\sum_{j\in 1\ldots N_{d}}k_{b}\left[1-\cos\left(\theta_{j}-\theta_{0}\right)\right] \end{array}$$

The single-bond polymerization/depolymerization model was first proposed by Bell [17]. Furthermore, Dembo et al. [18] developed a formula to calculate the polymerization/depolymerization rate, utilizing the Boltzmann distribution to account for affinity. We applied this model to delineate the polymerization/depolymerization occurrences between filaments and ACPs, as well as between filaments and membrane. A bond can form at a rate of $k_{on}$ when the distance between the free ligand and the receptor is sufficiently close. Conversely, if the length of the existing bond exceeds the breaking distance, it breaks at the rate of $k_{off}$:

(9) $$k_{on/off}=k_{on/off}^{0}e(\frac{-\sigma_{on/off}{\left(l-l_{0}\right)}^{2}}{2k_{B}T})$$

where *l* describes the instantaneous distance between the free ligand and the receptor, and $k_{on}^{0}$ and $k_{off}^{0}$ represent the bonding and dissociation rates when the distance between the free ligand and the receptor is $l_{0}$. The ACP particle forms a bond with neighboring filament particles with a probability of $P_{on}$:

(10) $$P_{on/off}=\left\{\begin{array}{ccc} 1-e^{-k_{on/off}\Delta t} & \; & l<d_{on/off}\\ 0 & \; & l\geq d_{on/off} \end{array}\right.$$

In DPD simulations, the above stochastic model has been widely utilized to investigate varying adhesion behaviors (bond formation) between healthy RBCs or between RBCs in sickle cell disease and macrophages in blood flow [19,20], the phenomenon of thrombus formation caused by platelet aggregation [21], and the adhesion dynamics of white blood cells in shear flow [22].

The process of assembling different components within the cell model was performed as follows: Non-intersecting filaments of varying lengths were randomly generated, and a specific concentration of ACPs was introduced into the cell space. This facilitated the random amalgamation of filaments and ACPs, forming a network characterized by substantial cross-linking. Subsequently, this network was integrated into the cell membrane model, culminating in the combination of membrane particles and cytoskeletal particles, mirroring the cross-linking process between filament and ACP particles. Finally, dihedral angle constraints were incorporated into the cytoskeleton model to achieve the ultimate model. These values were established by referencing previous work [12]. Furthermore, certain parameter adjustments were made to suit the requirements of the new model (Table 1).

#### 2.1.4. Particle-Tracking Rheology of Membrane

Particle-tracking rheology, which finds extensive application in biophysical settings, involves the introduction of small particles or droplets into fluids or cells to monitor their motion using lasers or cameras [26,27]. When measuring the modulus of the cell membrane, we analyzed it as a separate component and applied the particle-tracking rheology employed in the study of red blood cells to explore the membrane’s capacity to elastically store energy and dissipate stress as heat. A specific set of membrane particles was chosen, and their mean square displacements (MSDs) over time were recorded, expressed as

(11) $$\langle \Delta {r(t)}^{2}\rangle =\langle |r\left(t+t_{0}\right)-r\left(t_{0}\right){|}^{2}\rangle$$

where *t* is the time and $\langle |r\left(t+t_{0}\right)-r\left(t_{0}\right)|\rangle$ represents the MSDs of these particles.

To derive the storage modulus ($G^{\prime }$) and the loss modulus from the MSDs, we utilized an approximate method [28], outlined as:

(12) $$G^{*}\left(s\right)\approx \frac{k_{B}T}{\pi a\langle \Delta {r(t)}^{2}\rangle \Gamma \left[1+\chi (s)\right]}{|}_{s=\frac{1}{t}}$$

where *s* represents the Laplace frequency, $k_{B}$ represents the Boltzmann constant, *a* is the particle radius, Γ is a gamma function, and χ(s) represents the slope of $\langle \Delta {r(t)}^{2}\rangle$ in the log–log scale plot.

(13) $$\chi \left(s\right)\equiv \frac{d\ln\langle \Delta {r(t)}^{2}\rangle }{d\ln t}{|}_{s=\frac{1}{t}}$$

The storage modulus ($G^{\prime }$) and the loss modulus ($G^{\prime \prime }$) were computed using the following expressions:

(14) $$\begin{array}{c} G^{\prime }\left(s\right)=\left|G^{*}\left(s\right)\right|\cos\left(\frac{\pi \chi (s)}{2}\right)\\ G^{\prime \prime }\left(s\right)=\left|G^{*}\left(s\right)\right|\sin\left(\frac{\pi \chi (s)}{2}\right) \end{array}$$

Following the article [29], we performed curve fitting with a second-degree polynomial to analyze the slope at high frequencies in the log–log plot of frequency versus modulus. The same curve-fitting method was used in the of bulk rheology of the cytoskeleton as detailed in the next subsection.

#### 2.1.5. Bulk Rheology of Cytoskeleton

After conducting mechanical tests on the membrane, we proceeded with rheological studies concerning the cytoskeletal network. Through shear oscillation experiments, it was possible to measure the elastic modulus (*G*′) and viscous modulus (*G*″) of the cellular cytoskeleton. These moduli provided crucial information about the mechanical properties of the cell cytoskeleton, such as its resistance to stress and how it dissipated energy. Through these experiments, we could observe how the behavior of the cell cytoskeleton changed at different frequencies, thus gaining a deeper understanding of its response across different time scales. For shear rheology, a two-plate model was utilized to define the sample’s rheological behavior. This involved placing the sample between two parallel plates, with the lower plate being fixed and the upper plate being capable of moving parallel to the lower plate to exert a shear force [30].

(15) $$\gamma =\frac{s}{h}$$

where γ is the strain, *s* is the displacement of the upper plate, and *h* is the distance between two plates. The rate of shear is

(16) $$\dot{\gamma }=\frac{d\gamma }{dt}$$

Subsequently, we assessed the viscoelasticity of the sample via oscillatory shear tests. This entailed exerting sinusoidal shear strain at an oscillation frequency ($f_{s}$) and maximum strain ($\gamma_{0}$) onto the upper plate. When the strain was controlled within a narrow range, the stress response curve (τ) over time *t* resembled a sinusoidal curve, mirroring the applied sinusoidal strain’s frequency.

(17) $$\gamma \left(t\right)=\gamma_{0}\sin\left(2\pi f_{s}t\right)$$

Through the analysis of the stress–strain curve, we evaluated the viscoelastic moduli. The storage shear modulus ($G^{\prime }$) contributes to a material’s elasticity, while the loss modulus ($G^{\prime \prime }$) contributes to its viscosity [25].

(18) $$\begin{array}{c} |G^{*}\left(f_{s}\right)|=\frac{\left|\tau \right|}{\left|\gamma \right|}\\ |G^{\prime }\left(f_{s}\right)\left|=\right|G^{*}\left(f_{s}\right)|\cos\phi \\ |G^{\prime \prime }\left(f_{s}\right)\left|=\right|G^{*}\left(f_{s}\right)|\sin\phi \end{array}$$

where ϕ is the phase delay between strain and stress, whose value ranges between 0 and $\frac{\pi }{2}$. A value of 0 indicates no delay, signifying a purely solid elastic material, while a value of $\frac{\pi }{2}$ suggests a purely fluidic material.

In the simulation, we fully relaxed the polymerized cytoskeletal network under periodic boundary conditions. After stabilizing the system temperature, we immobilized the particles within 5% of the cytoskeletal network’s height at the bottom. By applying sinusoidal strain in the shear direction and varying the frequency, we obtained multiple sets of results after 10 cycles of strain application. Subsequently, the responsive shear stress curve was fitted to derive the amplitude and phase angle for computing the storage and loss modulus.

### 2.2. Cell Mechanics Models

#### 2.2.1. Indentation Model

An indentation model was devised within the DPD environment to investigate the mechanical attributes of the cell model, encompassing its two components. In this model, both the substrate and probe were constituted of DPD particles. Given that the probe’s stiffness exceeded that of the samples being measured, the simulation established the probe as a rigid body. Initially, the substrate was immobilized in its starting position, and the cell was positioned atop the substrate, permitting the cell to achieve complete relaxation. Simultaneously, a conical indenter was situated above the cell, inclined at an angle of 18°. Once the cell reached a fully relaxed state, constant speed was imparted to the probe, causing it to approach the cell. As the probe made contact with the cell, deformation ensued. Our recordings captured both the displacement of the probe and the load at the probe’s center of mass, forming the basis for a force–indentation curve. It is essential to note that the probe’s displacement was regarded as akin to the displacement of the piezoelectric scanner in the actual experimental setup. Adjustments were made to the membrane parameters, including the shear modulus ($\mu_{0}$), the bending stiffness ($k_{bending}$), and the maximum length ($l_{max}$). These adjustments aimed to explore their respective impacts on the force–indentation curves.

#### 2.2.2. Cell Microinjection Model

In the simulation, we replicated the cell microinjection DPD environment by mirroring the features of the real microinjection platform. This environment predominantly comprised a holding micropipette, a cell, and a microinjection micropipette. Both the holding and microinjection micropipettes were simplified as hollow cylinders constituted of DPD particles. The interaction between the particles of the holding and microinjection micropipettes was disregarded. Arranged along the *y*-axis, the holding and microinjection micropipette particles were fixed, allowing the cell to fully relax within this setup. To account for the intricate fluid dynamics surrounding and within the holding micropipette during aspiration, the negative pressure in the holding micropipette was simplified into a force field, attracting the membrane particles into the micropipette. As the aspiration process commenced, the cell approached the holding micropipette and underwent deformation due to the negative pressure’s influential force field. Once the aspiration process stabilized, we saved the current simulation state and initiated movement of the microinjection micropipette. Given that the cell membrane constituted a network comprising numerous WLC bonds, each bond was subject to a distinct stretching force during membrane deformation. We considered a bond to be broken when its length exceeded twice the equilibrium length. Upon this occurrence, the simulation halted, and the simulation data were exported.

We applied the continuum finite strain theory to compute cell deformation [31,32]. The distance vector between particle *i* and its neighboring particle *j* is given by

(19) $$\begin{array}{c} d_{ji}\equiv x_{j}-x_{i}\\ d_{ji}^{0}\equiv x_{j}^{0}-x_{i}^{0} \end{array}$$

where $d_{ji}^{0}$ represents the distance vector in the reference configuration, $d_{ji}$ represents the distance vector in the current configuration, and $N_{i}$ is the set of neighboring particles. To acquire a local affine transformation ($J_{i}$), we computed

(20) $$\begin{array}{c} d_{ji}\to d_{ji}^{0},\forall j\in N_{i}\\ \sum_{j\in N_{i}}\left|d_{ji}^{0}J_{i}-d_{ji}\right|=0 \end{array}$$

This can be expressed as

(21) $$J_{i}={\left(\sum_{j\in N_{i}}d_{ji}^{0T}d_{ji}\right)}^{-1}\sum_{j\in N_{i}}d_{ji}^{0T}d_{ji}$$

where $J_{i}$ is the deformation gradient tensor. By using polar decomposition, $J_{i}$ is decomposed into two matrices, namely, *R* and *U*. We define the strain (*E*) as

(22) $$\begin{array}{c} J_{i}=RU\\ E=U-I \end{array}$$

where *I* is the identity matrix. We interpret the main diagonal element of *E* as the tension-to-original-length ratio. To ascertain consistency between experimental and simulation results, we plotted the experimental strain as a histogram, denoted as distribution $P_{0}$. The simulation’s histogram was denoted distribution $P_{1}$. Metrics such as Jensen–Shannon (JS) divergence, the Jaccard similarity coefficient, and Bhattacharyya distance were employed to analyze the consistency degree between the experimental and simulation strain distributions.

Additionally, the DPD parameters (Table 2) used in simulation were cross-referenced with those in earlier work.

### 2.3. Experiments

#### 2.3.1. Cell Preparation

Ovaries were obtained from a slaughterhouse and promptly transported to our laboratory within 2 h of collection. They were stored in a thermos flask containing sterile saline in a temperature range of 35–37 °C. The experimental procedures were reviewed and approved by the Animal Care and Use Committee of Nankai University on 15 March 2015. Cumulus–oocyte complexes (COCs) ranging in diameter from 2 to 6 mm were extracted from follicles using a disposable syringe. These complexes were then cultured in a maturation medium within an incubator set to an atmosphere of 5% CO_2_ and 95% humidified air. The maturation medium consisted of TCM199 (with Earle’s Salts; Gibco, Grand Island, NY, USA) supplemented with 10% porcine follicular fluid (PFF), 0.1 mg/mL cysteine, 0.065 mg/mL penicillin, 10 ng/mL epidermal growth factor (EGF), 10 IU/mL equine chorionic gonadotropin (eCG; Intervet Pty. Ltd., Boxrneer, Australia), and 10 IU/mL human chorionic gonadotrophin (hCG; Intervet Pty. Ltd.).

#### 2.3.2. AFM Indentation

The AFM experiment was conducted using a Bioscope Resolve AFM system (Bruker, Billerica, MA, USA), which integrated an inverted optical microscope (Nikon Eclipse Ti2, Tokyo, Japan), a CCD camera, an SPM head functioning as a detector–probe assembly for accurate probe positioning, and a base plate. The probe featured an approximate tip radius of 20 nm and a length of approximately 175 μm, with a spring constant of 0.07 N/m. AFM indentation was performed on the oocytes positioned on a polylysine-coated slide. The imaging mode employed was PeakForce QNM in fluid, allowing for the direct control of the peak normal force and minimizing lateral force on the probe. Prior to the experiment, the probe’s spring constant underwent calibration using the AFM thermal noise module. The determination of the Young’s moduli of the oocytes was accomplished through the approach–retraction mode. During the approach stage, aided by the optical microscope, the probe gradually approached the cell surface. Upon contact with the cell surface, indentation occurred, causing the cantilever to deflect, reflecting the force applied to the probe. Subsequently, the retraction stage initiated as the deflection or the ramp size reached the specified value, leading to the probe being lifted away from the cell.

#### 2.3.3. Cell Microinjection

The cell microinjection experiments were carried out using a self-customized NK-MR601 micromanipulation robot system, assembled on an inverted microscope (Nikon ECLIPSE Ti2, Tokyo, Japan). This system featured an X-Y stage with a 100 mm × 100 mm moving range, a positioning resolution of ±0.1 μm, and a maximum speed of 2 mm/s. It comprised two self-developed X-Y-Z three-degree-of-freedom micromanipulation arms responsible for positioning the microinjection and holding micropipettes. As part of the experimental setup, a syringe provided aspiration pressure, delivering a negative pressure of −3∼0 kPa and a positive pressure of 0∼200 kPa. Visual inspection during microinjection was facilitated by a CCD camera (W-V460, Panasonic, Osaka, Japan), while image acquisition, data processing, and motion control were handled by a host computer. The holding pipettes utilized in the experiment were crafted from a borosilicate glass tube with an outer diameter of 1 mm and an inner diameter of 0.6 mm. These pipettes were manufactured by using an instrument (MODEL P-97, Sutter Instrument, Novato, CA, USA) and then forged into micropipettes with openings ranging from 50 to 80 μm by using a forging instrument (MF-900, Narishige, Amityville, NY, USA). The opening was smoothed by melting it with an alcohol lamp. The images of oocytes were acquired with a 10× objective lens at a spatial resolution of 0.625 μm. During the experimental process, the culture dish containing the cells was placed on the system stage. The microinjection micropipette and holding micropipette were positioned using the two operating arms, adjusted to the same focal plane. Negative pressure was applied through the holding micropipette to aspirate the cell, and the micropipette was injected at a controlled speed towards the cell’s central axis until the cell membrane was ruptured. The speed of the microinjection micropipette was set to 10, 30, and 50 μm/s. Visual data from the experiment were collected using a vision-based method to measure the strain inside the cells.

## 3. Results

### 3.1. Particle-Tracking Rheology of Membrane

**Figure 1:**
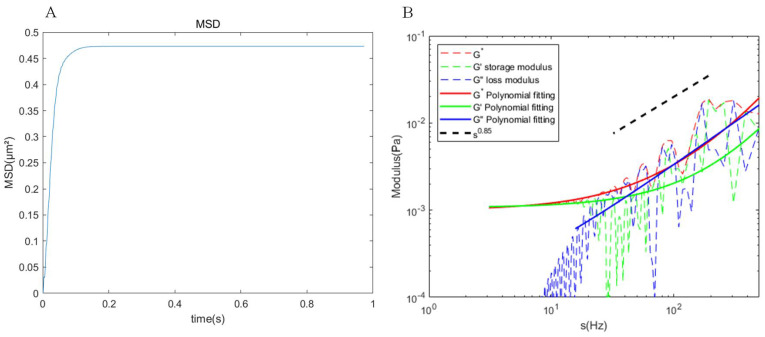
Storage and loss moduli of the cell membrane. (**A**) MSDs of the particles over time. (**B**) Storage modulus ($G^{\prime }$) and loss modulus ($G^{\prime \prime }$) calculated from the MSDs. The dashed line represents the simulation results, whereas the solid line represents the corresponding polynomial fitting.

In the simulation, we analyzed 40 particles on the cell membrane for particle-tracking rheology and monitored their mean square displacements (MSDs) over time. The particle movements on the cell membrane gradually stabilized, reaching a consistent state after 0.1 s (Figure 1A). Utilizing the MSD curve, we computed the storage modulus ($G^{\prime }$) and the loss modulus ($G^{\prime \prime }$) at various Laplace frequencies under room temperature conditions, as illustrated in Figure 1B. The results show a continuous increase in both storage and loss moduli with the increase in frequency. At lower frequencies, the elastic component (storage modulus) dominates, while at higher frequencies, the viscous component (loss modulus) surpasses the elastic contribution. Additionally, the storage and loss moduli of the cell membrane exhibit a weak power-law relationship at higher frequencies, approximated as $G∼s^{0.85}$. These observations collectively suggest that our cell membrane exhibits typical viscoelastic material properties.

### 3.2. Bulk Rheology of the Cytoskeletal Network

**Figure 2:**
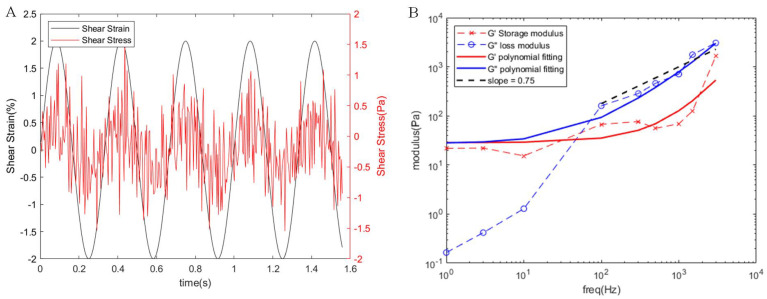
Bulk rheology of the cytoskeletal network. (**A**) Shear stress at a shear strain frequency of 3 Hz. It is evident that the resultant shear stress has the same periodicity as the applied shear strain, which was fitted with a sine function to obtain the amplitude and phase angle. (**B**) Storage and loss moduli of the cytoskeletal network at different frequencies. The x-shaped symbol indicates the storage modulus of the bulk rheology, which corresponds to the amount of energy that can be stored by the cytoskeletal network for reversible deformation. The o-shaped symbol denotes the loss modulus, which corresponds to the energy lost due to irreversible deformation. It can be observed that both moduli exhibit a weak power-law relationship with frequency at higher frequencies.

In the simulation, we subjected the three-dimensional cytoskeletal network to sinusoidal shear strain with a 2% amplitude across various frequencies ($f_{s}$). The cytoskeletal network’s elastic characteristics arise from the utilization of harmonic potential energy within the cross-linked filaments, whereas its viscous properties stem from both the dissipative force and the interlacing of cytoskeleton filaments. Analyzing stress curves generated at different shear strain frequencies allowed us to determine stress amplitude and phase differences concerning the applied shear strain (Figure 2A). Subsequently, we calculated the storage modulus and loss modulus of the cross-linked cytoskeletal network (Figure 2B).

At lower frequencies, the deformation behavior is predominantly influenced by the elastic properties. As the frequency escalates, the loss modulus surpasses the storage modulus at approximately tens of Hertz, indicating a transition in the cytoskeletal network towards fluidic properties. This shift might be attributed to the filaments being stretched for longer at lower frequencies, allowing them to have adequate time for recovery from deformation. During this phase, the energy loss due to particle friction within the network remains relatively minimal. However, at higher frequencies or shorter timescales, deformed filaments cannot adequately recover, leading to increased energy loss through particle friction and a consequent substantial increase in the loss modulus. Additionally, we observed a weak power-law relationship between the storage and loss moduli and the frequency $G∼{f_{s}}^{0.75}$, demonstrating the nonlinear mechanical properties of the cytoskeleton model. This characteristic evidences the viscoelastic semi-polymer network nature of the cytoskeleton model, aligning with the theoretical predictions outlined in [25,33,34,35,36].

### 3.3. Indentation Experiment

**Figure 3:**
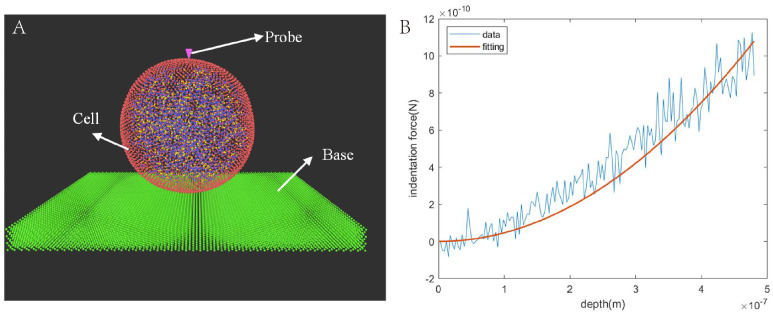
Indentation experiment. (**A**) Experimental setup. (**B**) Force–indentation curve. The Sneddon model was fit using a two-point method selecting the large-slope portion of the curve.

In the indentation simulation, we developed a probe comprising roughly 3000 DPD particles and directed it toward the cell (Figure 3A). Upon interaction between the probe and the cell, the cell initiated deformation, leading to the generation of elastic force that acted in opposition to the probe. As the probe progressed, the force applied to it increased due to the increasing number of WLC bonds engaged in elastic deformation. To fit the force–indentation curve using the Sneddon model, we selected a section of the curve (0.4–0.8 μm in Figure 3B) exhibiting a large slope. The Young’s modulus was determined to be 17.96 kPa, showing a slight deviation of approximately about 2% from the experimental result (17.63 ± 5.10 kPa) [37].

**Figure 4:**
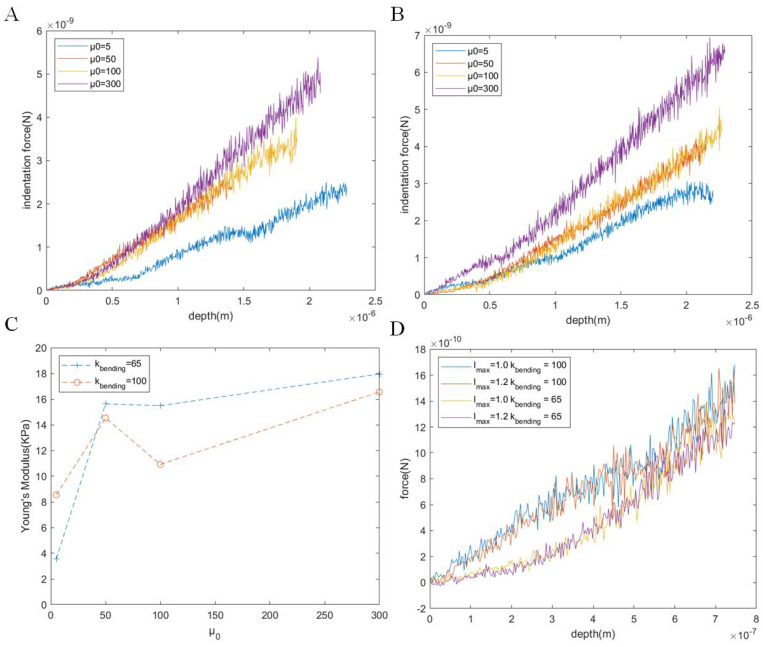
Change in the elastic properties of cells under different membrane parameters. Force–indentation curves for different values of $\mu_{0}$ = 5, 50, 100, or 300 and $k_{bending}=65$ (**A**) or $k_{bending}=100$ (**B**). (**C**) The relationship between Young’s modulus and $\mu_{0}$ for $k_{bending}$ = 65 or 100. (**D**) Force–indentation curves for different values of $l_{\max}$ and $k_{bending}$.

We further explored the effects of the shear modulus ($\mu_{0}$), the bending stiffness ($k_{bending}$), and the maximum length ($l_{\max}$) on the elastic properties of the cell membrane. We set the shear modulus to $\mu_{0}$ = 5, 50, 100, or 300 and conducted the indentation experiments, as shown in Figure 4. Under the influence of high shear modulus, the slope of the force–indentation curve tended to increase, meaning that the force imposed on the probe increased. The possible reason was that the shear modulus ($\mu_{0}$) affected the tensile properties of the cell membrane that related to the elastic force generated by the WLC bonds.

We further investigated the impact of the shear modulus ($\mu_{0}$), the bending stiffness ($k_{bending}$), and the maximum length ($l_{\max}$) on the elastic properties of the cell membrane. We set the shear modulus to $\mu_{0}$ = 5, 50, 100 or 300 and conducted the indentation experiments. Higher shear modulus values resulted in an increase in the slope of the force–indentation curve, indicating an elevated force applied to the probe. This effect may be attributed to the influence of the shear modulus on the tensile properties of the cell membrane, particularly concerning the elastic force produced by the WLC bonds.

In the simulation, we observed the impact of different bending stiffness values ($k_{bending}$) on the elastic properties of the cell. At shallow indentation depths (≤0.5 μm), a higher $k_{bending}$ corresponded to a greater indentation force. However, as the depth increased, this effect gradually diminished. The influence of $k_{bending}$ also varied concerning $\mu_{0}$. When $\mu_{0}\leq 50$, a larger $k_{bending}$ led to a higher Young’s modulus; however, this relationship was not evident when $\mu_{0}\geq 50$. We set $l_{\max}$ = 0.6, 0.8, 1.0, or 1.2 while maintaining the initial length of the WLCs constant to examine its role in the cell’s minor deformation. We observed nearly identical force–indentation curves for the same $k_{bending}$, indicating that the contribution of $l_{\max}$ to the elastic modulus was negligible during minor deformations.

### 3.4. Microinjection Experiment

**Figure 5:**
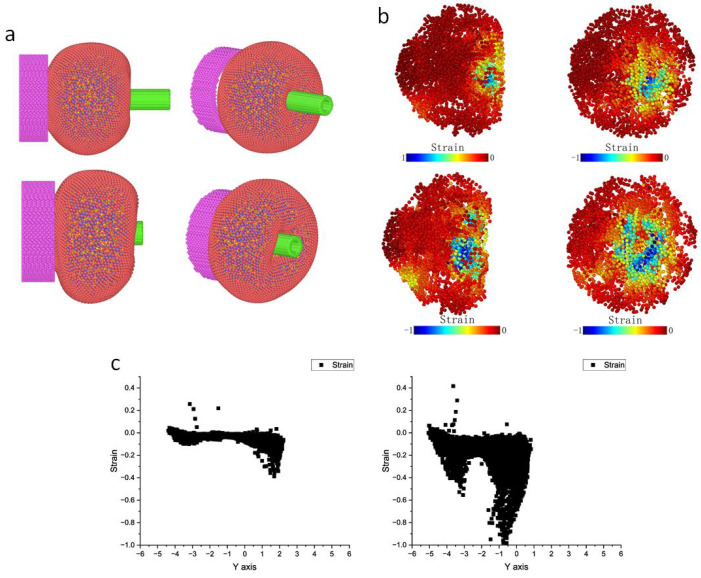
Microinjection experiment simulation. (**a**) Two stages (initiation of microinjection (upper) and some time later (below)) of microinjection. (**b**) Strain heatmaps of the two stages. (**c**) Magnitude distribution of strain in microinjection direction.

At the start of the simulation, the cell experienced deformation caused by a force field simulating negative pressure, resulting in a section of the cell being drawn into the aspiration micropipette, as depicted in Figure 5a. Once the aspiration stabilized, the microinjection micropipette commenced its movement toward the cell. Gradually, as the micropipette penetrated to a shallow depth, the entire cell underwent compression along the micropipette’s direction of movement. Continuing penetration led to substantial local deformation at the interaction point with the micropipette. Notably, the membrane in this area experienced noticeable stretching. As the micropipette reached a specific depth, the membrane bonds surpassed their designated length, resulting in the rupture of the cell membrane. We isolated the cytoskeleton component and specifically analyzed its strain during microinjection, as depicted in Figure 5b. Two image sets illustrate the strain magnitude at various penetration depths. The initial set of images portrays the cytoskeleton’s deformation at a shallow penetration depth, while the subsequent set illustrates deformation during deeper micropipette penetration. The regions of deformation, indicated by higher strain in blue, were primarily concentrated around the microinjection site. With increased penetration depth, the strain consistently increased, and the extent of deformation also increased progressively (Figure 5c).

**Figure 6:**
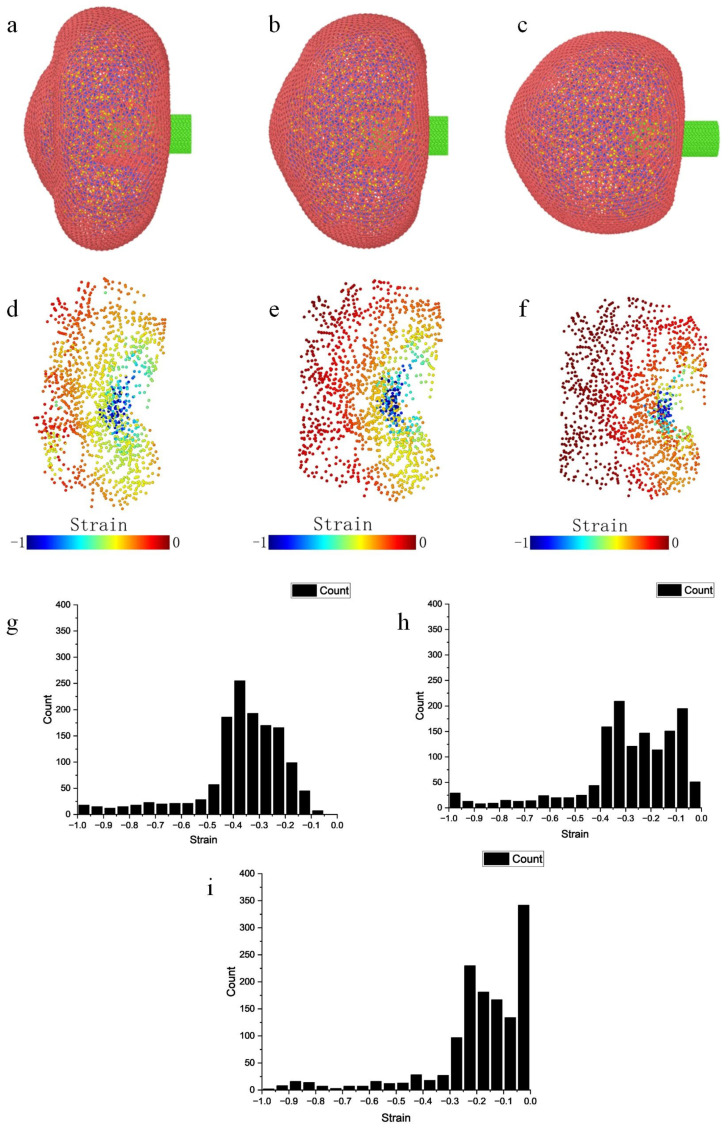
Analysis of microinjection simulation strain. (**a**,**d**,**g**) Strain generated at an injection speed of 2000 μm/s. (**b**,**e**,**h**) Stain generated at an injection speed of 6000 μm/s. (**c**,**f**,**i**) Strain generated at an injection speed of 10,000 μm/s.

We attribute the varying strain observed in cells responding to different micropipette injection speeds to the strain-rate dependency of biological tissue materials. At lower speeds, cells showed more pronounced deformation, suggesting higher flexibility of biological structures when subjected to slower impacts. Conversely, at higher speeds, cells exhibited rigid characteristics, indicating a stiffening response. This dual behavior reflects their nonlinear response to external stimuli at different speeds. To further examine the strain-rate dependence phenomenon, we conducted three sets of experiments. In the second and third sets, the micropipette penetration speeds were three times and five times that of the first set, respectively (Figure 6). As the penetration speed increased, the extent of cell deformation upon contact with the micropipette decreased. For instance, in the third experiment set, the cell region farther from the micropipette showed minimal deformation. Additionally, the degree of deformation at the moment of rupture decreased at higher speeds, resulting in a smaller proportion of large strain.

**Figure 7:**
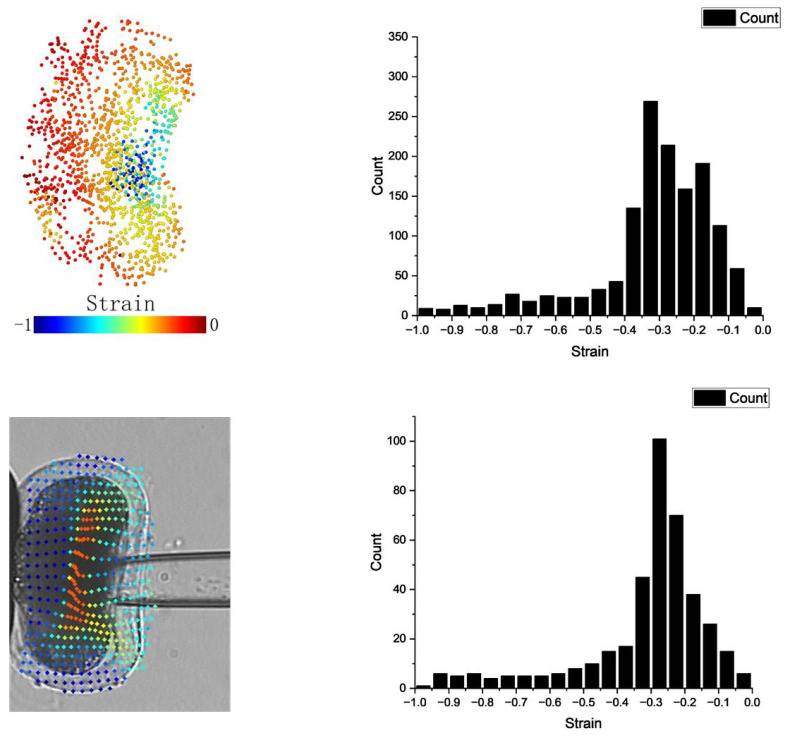
Comparison of strain calculation between simulation (**above**) and actual experiment (**below**). The penetration speed was 10 μm/s for both the simulation and actual experiment. In the simulation, we selected the cross-section near the center of the cell and mapped the points onto a two-dimensional plane. A histogram was used as a metric for comparing the results.

It is important to note differences in strain calculation methods between the simulation and the actual experiment. In the simulation, strain was directly computed from a three-dimensional point cloud, whereas in the experiment, strain was derived from two-dimensional images. Additionally, the simulation randomly selected cytoskeleton particles for strain calculation, while in the images, the selected feature points were uniformly distributed (by using optical flow estimation). Within the three-dimensional point cloud, we opted for the cross-section near the cell’s center and projected the points onto a two-dimensional plane to compute strain in the penetration direction (Figure 7). To compare simulation and experimental results for cells penetrated at a speed of 10 μm/s, we utilized a histogram as a metric. By comparing the strain distributions, we observed a prominent peak in the low-strain region within the range of [−0.4, −0.2]. Upon plotting the strain histogram, we used the metrics Jaccard similarity coefficient, Bhattacharyya distance, and JS divergence to gauge similarity between simulation and experimental distributions. The Jaccard similarity coefficient yielded a value of 0.655, while Bhattacharyya distance and JS divergence were 0.0272 and 0.0267, respectively. These values suggest that our model effectively captured deformation patterns akin to those observed in the experiments.

## 4. Discussion

Micromanipulation induces intracellular strain altering cell viscoelasticity, potentially reducing developmental potential. Optimizing micromanipulation procedures requires an interactive cell micromanipulation model for intracellular strain analysis. Here, we used the DPD method to construct a cell model encompassing cell membranes and cytoskeleton. To validate this modeling approach, we conducted mechanical tests: particle-tracking microrheology for the cell membrane and shear oscillation for the cytoskeleton. Both experiments generated storage and loss modulus curves at varying frequencies, revealing time-dependent viscoelasticity and a weak power-law relationship between modulus and frequency. Differing from our prior work, this study adopted a higher-precision cell membrane model and increased the density of the cytoskeleton. The cytoskeletal network had stronger connectivity and could more effectively simulate cell deformation and mechanical response. Meanwhile, we carried out more experiments to study the mechanical properties of each component. We studied the viscoelastic behavior of the cell membrane by analyzing the particle-tracking rheology and verified the power-law dependency of the cytoskeleton model on frequency through the oscillatory shear test. In addition, we discussed the mechanical response of cells with different parameters, such as the $l_{max}$ of WLCs, the shear modulus (μ), and the bending stiffness ($k_{bending}$).

We established a nanoindentation simulation, evaluating overall cell elastic properties. By plotting force–indentation curves and applying the Sneddon model, we obtained Young’s modulus (17.96 kPa vs. 17.63 kPa for simulation vs. experiment, respectively). We examined which membrane parameters had the most significant impact on the elastic properties by adjusting their value and observing the changes in the force–indentation curves and Young’s moduli.

Additionally, a microinjection experiment studied cell deformation during injection. Using finite strain theory, we calculated normal strain in the injection direction, quantitatively comparing results with the actual experiment. The investigation of micropipette injection speed’s influence on intracellular strain revealed varied cell deformation patterns. These variations correspond to typical viscoelastic traits of biological tissues, demonstrating strain rate dependency in their responses. Our model replicated the observed phenomena, showing increased injection speed correlated with smaller strain at membrane rupture.

In this cell model, the construction of the cell membrane followed our established method, whereas the cell cytoskeleton underwent improvements from our previous work. Both components remained phenomenological models; for instance, the cell membrane did not consider biological macromolecules on its surface. The randomly generated coarse-grained cytoskeletal filaments exhibited non-uniform spatial distribution, resulting in inconsistent mechanical properties across various locations. In our future studies, we aim to model the cell cytoskeleton at a finer scale and integrate it into our mesoscopic model after coarse-graining. This approach will enhance the accuracy of cytoskeleton modeling at the mesoscopic level.

Our upcoming work involves additional microscale experiments to further validate our models, including microfluidic experiments and cell transport studies. We plan to delve into changes in viscoelasticity linked with variations in cytoskeleton parameters, such as actin filament concentration, filament length, cross-linker concentration, and polymerization parameters. Furthermore, we aim to refine our cell modeling by incorporating additional components, such as cell nuclei and polar bodies.

## 5. Conclusions

In this study, we developed a cell model comprising a triangular network-like cell membrane and cross-linked cytoskeletal chains. We separately explored the mechanical properties of the membrane and cytoskeletal network through a series of simulated mechanical testing experiments. By using particle-tracking rheology, we recorded the mean square displacements of the membrane particles over time and subsequently computed the storage and loss moduli of the membrane. Additionally, we conducted a double-plate oscillatory shear experiment on the cytoskeletal network to analyze its storage and loss moduli. The results demonstrate that both the membrane and cytoskeleton exhibit viscoelastic characteristics, given their storage and loss moduli dependencies on frequency ($s^{0.85}$ and $s^{0.75}$). Furthermore, we conducted indentation and microinjection simulations to mimic actual experiments and analyze the mechanical properties of whole cells. In the indentation experiment, we recorded probe displacement and load at the probe’s center of mass, generating force–indentation curves. We varied membrane parameters and examined their effects on force–indentation curves. In the microinjection experiment, we studied cell deformation and intracellular strain distribution under varying microinjection speeds. We compared the resulting strain distribution with visual inspection data from the actual experiment by using metrics like Jensen–Shannon (JS) divergence, the Jaccard similarity coefficient, and Bhattacharyya Distance.

## Figures and Tables

**Table 1:** Parameters used in the simulation.

Notation	Parameter	Physical Value	Simulation Value	Source
$r_{c}$	Cell radius	120 μm	8	[12]
$k_{B}T$	Energy scale	4.1164 × 10^−21^ J	1	[12]
$k_{v}$	Volume constant	-	7.5 × 10^3^	[12,22,23,24]
$k_{a}$	Area constant	-	7.5 × 10^3^	[12,22,23,24]
$k_{d}$	Local area constant	-	300	[12,16]
$N_{a}$	Actin number	-	2698	-
$L_{f}$	Filament length	-	0.5–2.5	-
$N_{af}$	Actin number on filament	-	3–11	-
$k_{f}$	Filament spring constant	0.092 N/m	8 × 10^4^	[12,22,23]
$k_{fbend}$	Filament bending stiffness	4.025 × 10^−16^ J	350	[12,22,23]
$N_{A}$	ACP number	-	1000	-
$k_{A}$	ACP spring constant	0.0092 N/m	8 × 10^3^	[12,22,23,25]
$k_{f-A}$	ACP–filament bending stiffness	6.325 × 10^−16^ J	550	[12,16,22,23]
$k_{t}$	ACP–filament torsion stiffness	4.7 × 10^−16^ J	470	[12,16,22,23]
$k^{0}$	Zero-force unbinding rate	78 s^−1^	26 × 10^−4^	[12,16,23]
$d_{on}$	Binding distance	-	0.25	[12,23]
$d_{off}$	Unbinding distance	-	0.25	[12,23]
$\sigma^{off}$	Switch strength for cytoskeleton	3.5 × 10^−4^ μm	3.5 × 10^−4^	[12]
$\sigma^{off}$	Switch strength for cell	10^−4^ μm	10^−4^	[12]

**Table 2:** Conservative parameters ($a_{ij}$), amplitudes of dissipative force (γ), and cutoff radius ($R_{c}$). The interaction between the holding micropipette and the microinjection micropipette is ignored.

	Membrane	Actin	ACP	Probe	Substrate	Holding Micropipette	Microinjection Micropipette
1	100/45/0.5	100/45/0.5	100/45/0.5	100/65/0.25	100/65/0.25	30/65/0.5	100/65/0.25
2	-	100/65/0.25	100/65/0.25	100/65/0.25	100/65/0.25	100/65/0.5	100/65/0.25
3	-	-	100/65/0.25	100/65/0.25	100/65/0.25	100/65/0.5	100/65/0.25

## Data Availability

The data that support the findings of the study are available upon request.
